# Synthesis and Characterization of Carbon-11 Labeled Iloperidone for Imaging of α_1_-Adrenoceptor in Brain

**DOI:** 10.3389/fmolb.2020.586327

**Published:** 2020-09-24

**Authors:** Yulong Xu, Yanli Wang, Hao Wang, Changning Wang

**Affiliations:** Athinoula A. Martinos Center for Biomedical Imaging, Department of Radiology, Massachusetts General Hospital, Harvard Medical School, Charlestown, MA, United States

**Keywords:** α_1_-adrenoceptor, neuronal diseases, PET/CT imaging, [^11^C]iloperidone, brain exposure

## Abstract

α_1_-Adrenoceptor is implicated in numerous neuronal diseases. The development of new modulators targeting this receptor as well as the investigation of the role of α_1_-adrenoceptor in healthy and disease conditions, however, is hindered by the lack of specific positron emission tomography (PET) radiotracers. Iloperidone shows a high binding affinity to α_1_-adrenoceptor and moderate selectivity over other brain receptors. We report herein the synthesis and characterization of carbon-11 labeled iloperidone for imaging of α_1_-adrenoceptor in brain. The radiolabeling of [^11^C]iloperidone was carried out conveniently in one step by treating precursor with [^11^C]CH_3_I in DMF in the presence of K_2_CO_3_. Then, [^11^C]iloperidone was purified by semi-preparative HPLC, and characterized in C57BL/6 mice using PET/CT scanning. The desired product [^11^C]iloperidone was obtained in an average decay corrected radiochemical of 12% (*n* = 3) and over 99% radiochemical purity. The average molar radioactivity was 357 GBq/μmol with total synthetic time of 35–40 min. PET/CT scanning in C57BL/6 mice showed favorable pharmacokinetic properties and high brain exposure of [^11^C]iloperidone. Blocking experiments by pretreatment with the unlabeled iloperidone showed the significant blocking effects with about 25% reduction in brain uptake. These results suggested that [^11^C]iloperidone can serve as a lead compound for the further development of specific radiotracers for PET imaging of α_1_-adrenoceptor in brain clinically.

## Introduction

The α_1_-adrenoceptor is G protein-coupled receptor, widely distributed in both the peripheral and the central nervous system (CNS; Gross et al., 1989; Zhong and Minneman, 1999). In human CNS, α_1_-adrenoceptor is mainly localized in the cerebral cortex, thalamus and hippocampus, while moderately in striatum, and cerebellum (Palacios et al., 1987; Kalaria, 1989). Over the past decade, α_1_-adrenoceptor has gained interest due to its biological functions in regulating both positively motivated behaviors and stress reactions (Selken and Nichols, 2007; Stone et al., 2007). The α_1_-adrenoceptor has been implicated in numerous neuronal diseases, such as Alzheimer’s disease, Parkinson’s disease, schizophrenia, substance abuse, and affective disorders (Maletic et al., 2017; Wu et al., 2017; Akinaga et al., 2019; Datta et al., 2019). Consequently, a great deal of interests lay in the development of α_1_-adrenoceptor modulators, which has opened a new avenue for identifying novel CNS disease modifying therapeutics.

**FIGURE 1:**
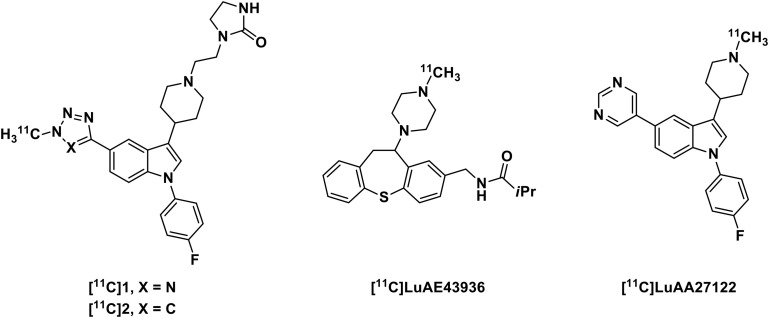
Structures of some representative α_1_-adrenoceptor PET radiotracers.

Positron emission tomography (PET) is a fully translational imaging technique, allowing the detection of picomolar sensitivity (Bergstroöm et al., 2003). An α_1_-adrenoceptor specific PET radiotracer would be highly beneficial in the development of novel α_1_-adrenoceptor modulators as well as in understanding the role of α_1_-adrenoceptor in progression of CNS disease. Accordingly, substantial research effort has been devoted to the development of efficient PET radiotracer for probing α_1_-adrenoceptor (Figure 1; Airaksinen et al., 2013; Risgaard et al., 2013). Among these PET ligands, [^11^C]LuAA27122 exhibited favorable blood-brain barrier (BBB) penetration, binding at α_1_-adrenoceptor rich regions in cynomolgus monkey brain while lacking specificity on α_1_-adrenoceptor, which has been confirmed through autoradiography studies *in vitro*. No specific α_1_-adrenoceptor PET radiotracer with sufficient brain exposure in clinic has hampered investigation of the role of α_1_-adrenoceptor in healthy and disease conditions as well as the development of new modulators targeting this receptor.

As our continuing interest in PET radiotracers for CNS (Wang et al., 2015, 2017; Chen et al., 2019), we decided to pursue an efficient α_1_-adrenoceptor PET ligand with desirable binding affinity and selectivity, as well as suitable metabolic stability. Iloperidone, an effective antipsychotic drug in clinic, demonstrates a high binding affinity to α_1_-adrenoceptor (IC_50_ = 0.4 nM) and sufficient selectivity over other brain receptors, such as serotonin 5-HT_2_ (IC_50_ = 9.0 nM), 5-HT_1__A_ (IC_50_ = 210 nM), dopamine D_1_ (IC_50_ = 750 nM) and D_2_ (IC_50_ = 110 nM) receptors (Strupczewski et al., 1995). Functionally, iloperidone shows antagonistic properties at dopamine D_2_ and serotonin 5-HT_2_ receptors, presumably acting as an antagonist at α_1_-adrenoceptor, which may contribute to its antipsychotic effects (Stahl, 2013). Recently, Joshi’s group demonstrated the repurposing of iloperidone on anti-hypertensive and ocular hypotensive activity in animals, which was related to its effect on peripheral α_1_-adrenoceptor (Joshi et al., 2020). As a result, the biological properties of iloperidone support its carbon-11 labeled “hot” compound as a promising PET radiotracer for α_1_-adrenoceptor in the human brain. Herein, we describe our results on the radiosynthesis of [^11^C]iloperidone as well as PET imaging evaluation in mice for α_1_-adrenoceptor.

## Materials and Methods

### General

All commercially available reagents were used without further purification unless otherwise stated. Iloperidone was purchased from Fisher Scientific. Analytical thin layer chromatography (TLC) was performed using Silica Gel GF254 plates (Merck Millipore co,.ltd, 0.2 mm thick). Compounds were purified using CombiFlash Rf 150 (Teledyne ISCO co,.ltd). ^1^H spectra was recorded on Bruker 500 MHz. Chemical shifts in ^1^H NMR spectra was reported in parts per million (ppm) on the δ scale from an internal standard of CDCl_3_ (7.26 ppm). Data were reported as follows: chemical shift (δ ppm), multiplicity (s = singlet, d = doublet, t = triplet, q = quartet, m = multiplet, and br = broad), coupling constant in hertz (Hz), and integration. MS data was recorded on Agilent Technologies 6310 quadrupole mass spectrometer.

All mouse studies were carried out at Massachusetts General Hospital (PHS Assurance of Compliance No. A3596-01). The Subcommittee on Research Animal Care (SRAC) serves as the Institutional Animal Care and Use Committee (IACUC) for the Massachusetts General Hospital. SRAC reviewed and approved all procedures detailed in this paper. All mice were socially housed in cages appropriate for the physical and behavioral health of the individual animal and were given unlimited access to food and water, with additional nutritional supplements provided as prescribed by the attending veterinary staff.

### Preparation of Precursor

A mixture of iloperidone (10 mg, 0.02 mmol) and concentrated H_2_SO_4_ (1 mL) was stirred at 65°C for 23 h. The cooled reaction was poured into 1 *g* of ice and was stirred vigorously for 10 min. The aqueous mixture was extracted with CH_2_Cl_2_ and the resultant CH_2_Cl_2_ extract was washed with 5% sodium hydroxide. The basic phases were combined and washed with CH_2_Cl_2_. The aqueous mixture was cooled in an ice bath and concentrated hydrochloric acid was added until a precipitate formed. The crude product was purified via flash column chromatography on silica gel to provide the precursor in 85% yield (7 mg). ^1^H NMR (500 MHz, CDCl_3_) δ 7.98 (s, 1H), 7.57 (d, J = 2.1 Hz, 1H), 7.42 (dd, J = 8.3, 2.1 Hz, 1H), 7.23 (dd, J = 8.5, 2.1 Hz, 1H), 7.08 (td, J = 8.9, 2.2 Hz, 1H), 7.02 (d, J = 8.2 Hz, 1H), 4.09 (t, J = 5.2 Hz, 2H), 3.29 (s, 3H), 2.74 (s, 2H), 2.56 (s, 3H), 2.36 (s, 2H), 2.05 (s, 4H), 1.30 (s, 1H), 1.25 (s, 2H), 1.24 (s, 1H). MS (ESI) m/z 413.3 [M + H]^+^.

### Radiosynthesis of [^11^C]Iloperidone

The radiosynthesis of [^11^C]iloperidone was carried out according to the procedure described previously by our group (Chen et al., 2019). Briefly, [^11^C]CO_2_ was obtained via the ^14^N (*p*, α) ^11^C reaction on nitrogen with 2.5% oxygen, with 11 MeV protons (Siemens Eclipse cyclotron), and trapped on molecular sieves in a TRACERlab FX-MeI synthesizer (General Electric). [^11^C]CH_4_ was obtained by the reduction of [^11^C]CO_2_ in the presence of Ni/hydrogen at 350°C, which re-circulated through an oven containing I_2_ to produce [^11^C]CH_3_I via a radical reaction.

The prepared [^11^C]CH_3_I was trapped in anhydrous DMF (300 μL) containing precursor (1.0 mg) and K_2_CO_3_ (5.0 mg). The reaction vessel was heated at 80°C and kept there for 3 min. The radioactive mixture containing [^11^C]iloperidone was quenched by addition of an HPLC mobile phase (0.7 mL) and then applied to a reverse phase semipreparative HPLC (Phenomenex Gemini-NX 5u C18 110A, 250 × 10 mm, 5.0 mL/min, 65% H_2_O + 0.1% TFA/35% CH_3_CN). A radioactive fraction having a retention time of 10 min was collected in a flask, and diluted in water (30 mL). The final product was reformulated by loading onto a solid-phase exchange (SPE) C-18 cartridge (Waters WAT020515 Sep-Pak Plus Short C18), rinsing with water (4 × 5 mL), eluting with EtOH (0.3 mL), and diluting with saline (2.7 mL). The chemical and radiochemical purity of the final product was tested by analytical HPLC (VARIAN Puruit XRs 5 C18, 150 × 4.6 mm), eluting with a gradient of 10–90% CH_3_CN in H_2_O of 0.1% TFA, at a flow rate of 1.5 mL/min. Confirmation of the identity of [^11^C]iloperidone was achieved by co-injection with iloperidone as reference standard. For the determination of molar activity, mass (μmol) of [^11^C]iloperidone with a known radioactivity was determined by HPLC comparison of UV absorbance at 254 nm with those of known concentrations of non-radioactive iloperidone.

### PET/CT Acquisition and Post Processing

Positron emission tomography/CT imaging was performed in anesthetized (isoflurane) C57BL/6 mice (20–25 *g*, female; *n* = 2 for baseline, and *n* = 2 for blocking) to minimize discomfort. Highly trained animal technicians monitored animal safety throughout all procedures. The mice were fixed on the bed of a Triumph Trimodality PET/CT scanner (Gamma Medica, Northridge, CA, United States) in the prone position, and injected with [^11^C]iloperidone (150–200 μL, ∼7.4 MBq) via a lateral tail vein catheterization at the start of PET acquisition. For blocking studies, iloperidone (5 mg/kg, iv) was injected at 10 min prior to [^11^C]iloperidone injection. Dynamic PET acquisition lasted for 60 min and was followed by CT for anatomic co-registration. PET data were reconstructed using a 3D-MLEM method resulting in a full width at half-maximum resolution of 1 mm. Reconstructed images were exported from the scanner in DICOM format along with an anatomic CT. These files were imported to AMIDE software (version 1.0.4).

### PET/CT Image Analysis

Positron emission tomography images were analyzed using the freely available AMIDE software (version 1.0.4). Volumes of interests (VOIs) were drawn manually as spheres guided by high resolution CT structural images and summed PET data, with a radius no less than 1 mm to minimize partial volume effects. Time-activity curves (TACs) were exported in terms of decay corrected activity at specified time points with gradually increasing intervals. The TACs were expressed as the percentage of injected dose per gram (% ID/g).

## Results and Discussion

**SCHEME 1:**
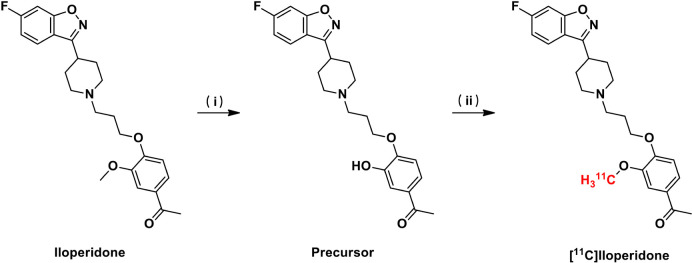
Radiosynthesis of [^11^C]iloperidone. Reagents and conditions: (i) H_2_SO_4_, 65°C, yield 85%. (ii) [^11^C]CH_3_I, K_2_CO_3_, DMF, 80°C, yield 12% (decay corrected).

As shown in Scheme 1, the radiolabeling of [^11^C]iloperidone was carried out conveniently in one step. The precursor was prepared by treating iloperidone in concentrated H_2_SO_4_ at 65°C for 23 h in the yield of 85%. The [^11^C]CH_3_I reacted with the precursor in DMF at 80°C for 3 min in the presence of K_2_CO_3_, and the radioactive mixture was purified by semi-preparative HPLC, eluting with 0.1% trifluoroacetic acid solution of water and acetonitrile (65:35) at a flow rate of 5 mL/min. The fraction containing the product was collected at around 10 min and passed through a C_18_ Sep-Pak column, then eluted with 20 mL water. The desired product [^11^C]iloperidone was obtained by eluting 0.3 mL of ethanol through the C_18_ Sep-Pak column in 2.7 mL of sterile saline, resulting the final formulation with 12% radiochemical yield (*n* = 3, decay corrected) and over 99% radiochemical purity. The total time of [^11^C]iloperidone synthesis was 35–40 min with a molar activity of 357 GBq/μmol.

**FIGURE 2:**
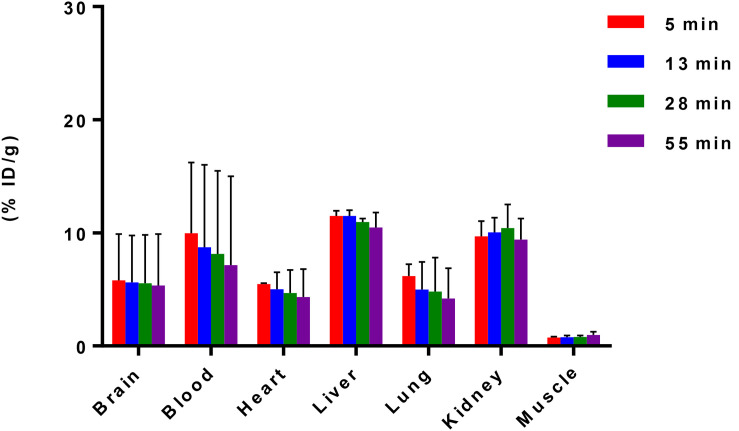
Biodistribution of [^11^C]iloperidone in selected organs of C57BL/6 mice (20–25 *g*, female, and *n* = 2). Data are expressed as the percentage of injected dose per gram (% ID/g).

After reliable and successful radiosynthesis of [^11^C]iloperidone, PET/CT scanning was performed in C57BL/6 mice (20–25 *g*, female, *n* = 2) to evaluate its biodistribution, which can quantify accumulation and determine metabolic pathways of [^11^C]iloperidone in different organs. As shown in Figure 2, [^11^C]iloperidone showed high initial uptakes in liver and kidney (11.49% ID/g and 9.70% ID/g at 5 min post injection, respectively), and subsequent slow clearance (10.48% ID/g and 9.42% ID/g at 55 min post injection, respectively), demonstrating that [^11^C]iloperidone was mainly excreted through bile and urine. However, very low uptake in muscle were observed for [^11^C]iloperidone (0.75% ID/g at 5 min post injection). The blood displayed the highest uptake with 9.96% ID/g at 5 min, followed by lung with 6.18% ID/g at 5 min, brain with 5.79% ID/g at 5 min, and heart with 5.48% ID/g at 5 min. Notably, [^11^C]iloperidone had a relatively slow clearance rate in blood (7.15% ID/g at 55 min post injection), indicating a long-time blood level.

**FIGURE 3:**
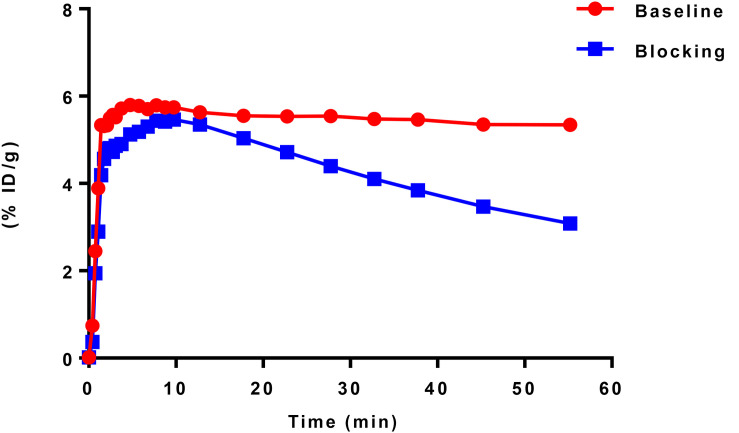
TACs of baseline (20–25 *g*, female, and *n* = 2) and blocking (20–25 *g*, female, and *n* = 2) experiments in C57BL/6 mouse brains. Data are expressed as the percentage of injected dose per gram (% ID/g).

**FIGURE 4:**
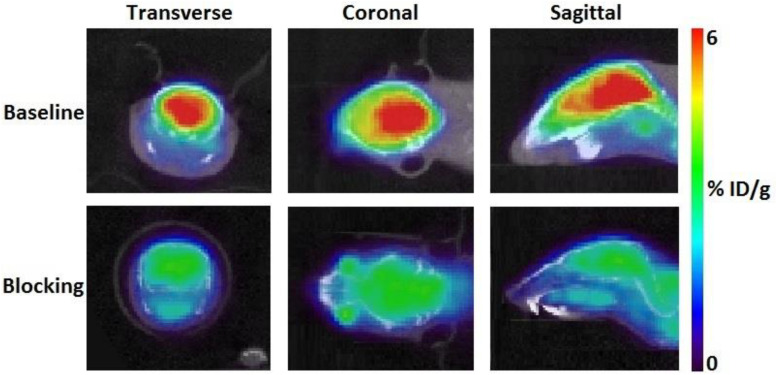
Transverse, coronal and sagittal PET/CT images of [^11^C]iloperidone in baseline **(above)** and blocking **(below)** experiments.

According to TAC in mouse brain, [^11^C]iloperidone exhibited high brain exposure with uptake reaching a plateau at 5 min post injection, followed by a very slow washout (Figure 3). These results demonstrated that [^11^C]iloperidone might perform irreversible kinetics toward α_1_-adrenoceptor in brain. Additionally, the regional distribution of [^11^C]iloperidone in brain was homogenous (Figure 4), demonstrating no evident regional binding difference, which might attribute to the moderate selectivity of iloperidone on α_1_-adrenoceptor. To test the specificity of [^11^C]iloperidone toward α_1_-adrenoceptor in brain, we carried out blocking experiments by pretreatment of “cold” iloperidone (5 mg/kg) in C57BL/6 mice (20–25 *g*, female, and *n* = 2). The blocking TAC showed that the binding of [^11^C]iloperidone was blocked in mouse brain (Figures 3, 4), and the brain uptake was about 25% reduced based on the area under curve (AUC, 13 min to 55 min) comparing with baseline.

## Conclusion

In summary, we have synthesized and characterized carbon-11 labeled iloperidone for imaging α_1_-adrenoceptor in brain. The radiolabeling of [^11^C]iloperidone was carried out conveniently in one step with good radiochemical yield and high radiochemical purity. The PET imaging studies in C57BL/6 mice demonstrated that [^11^C]iloperidone displayed favorable pharmacokinetic properties and exhibited high brain exposure. Blocking experiments by pretreatment with the unlabeled iloperidone showed the significant blocking effects with about 25% reduction in brain uptake. Despite of the promising preclinical profile in C57BL/6 mice, the clinical use of [^11^C]iloperidone might be hindered by its moderate specificity on α_1_-adrenoceptor. The efficient brain penetration of [^11^C]iloperidone, however, suggested that it can serve as a lead compound for the further structure activity relationship (SAR) exploration and optimization studies. We are continuing in this vein and will report our findings in due course.

## Data Availability Statement

All datasets presented in this study are included in the article/supplementary material.

## Ethics Statement

The animal study was reviewed and approved by Subcommittee on Research Animal Care at Massachusetts General Hospital.

## Author Contributions

YX and CW initiated this research project. YX, HW, and YW processed the data and performed the data analysis. YX and CW designed, wrote, and reviewed the manuscript. All authors read and approved the final manuscript.

## Conflict of Interest

The authors declare that the research was conducted in the absence of any commercial or financial relationships that could be construed as a potential conflict of interest. The handling editor declared a shared affiliation, though no other collaboration, with the authors.
